# Sucking Pads: A Report of Two Newborns

**DOI:** 10.7759/cureus.10904

**Published:** 2020-10-11

**Authors:** Vijayakumary Thadchanamoorthy, Markandu Thirukumar, Kavinda Dayasiri

**Affiliations:** 1 Clinical Sciences Department, Faculty of Health Care Sciences, Eastern University, Batticaloa, LKA; 2 Paediatrics Department, Base Hospital, Mahaoya, LKA

**Keywords:** sucking pad, vermillian boarder, brainstem, sucking reflex, newborn, preterm babies, lips, non-consanguineous parents, breastfeeding, oral thrush

## Abstract

The sucking pad is a hyperkeratotic thickening of the lips of a neonate. It might present either at birth or develop later in the neonatal period. It indicates that the child has effective sucking. We present two babies who developed the peeling of the lips during the neonatal period. It was mistreated as various conditions. Ultimately, it was diagnosed as a sucking pad, and the parents were reassured that it was a benign lesion.

## Introduction

The sucking pad is also known as sucking callus. It is described as a combination of intracellular edema and hyperkeratotic thickening of the lips [1]. It occurs at the inner aspect of the vermillion border of the lips of the newborn due to friction in terms of effective sucking [2]. The baby first develops sucking reflex at the gestational age of 25 weeks in utero as a result of a primitive sucking reflex which is mediated by the brainstem [2]. It can be seen in both full-term and preterm babies [3]. The presence of a sucking pad indicates that the baby has intact motor neuron function and effective sucking. It is considered as a neonatal screening tool [1-2]. Although it has been described in various neonatology and pediatric textbooks, there was limited literature found in the review of the reported literature [1-2]. We report two newborns who presented with sucking pads following various unsuccessful treatments for this benign condition.

## Case presentation

Case 1

A three-week-old baby was brought to an obstetrician as the baby had a lesion on the lips since two weeks of age and the mother also had sore nipples and vaginal bleeding. The lesion in the baby’s lip was neither painless nor did it interfere with sucking. This was referred to the paediatrician for further evaluation. The baby had good sleep in-between feeding and gained weight appropriately. The baby was not responding to either routine home remedies or treatment of the general practitioner (GP). He was the third-born male child of non-consanguineous parents. He was born by spontaneous vaginal delivery at term with a birth weight of 3 kg. The baby had established breastfeeding and was discharged on the same day as the neonatal examination had been normal. Bacillus Calmette-Guérin (BCG) vaccination was given before discharge. Since then, the baby had regular on-demand breastfeeding, and bowel and urinary frequency were within normal limits.

**Figure 1:**
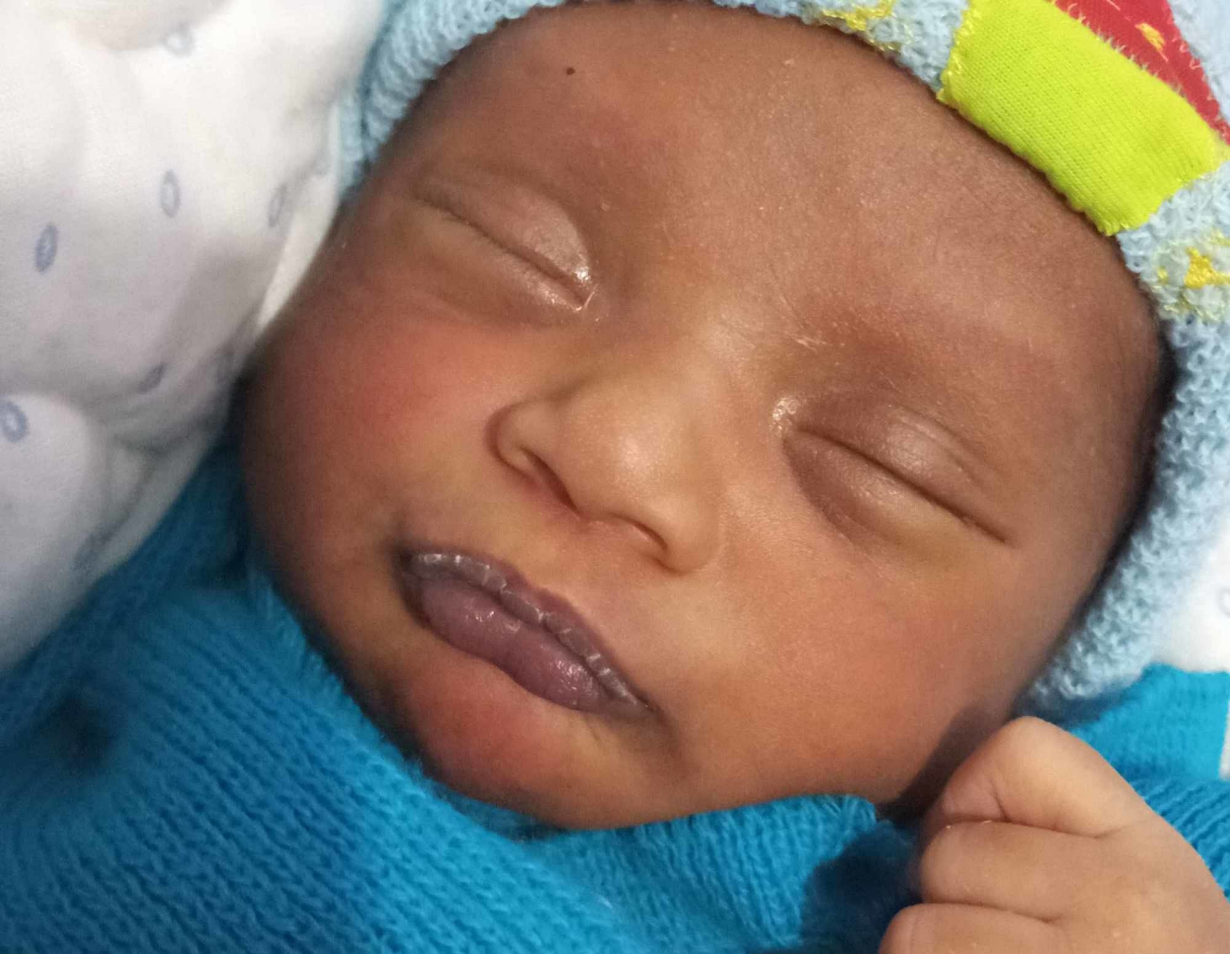
Desquamating sucking pad

Examination revealed a well-looking, active, pink, and well-hydrated baby. The baby had weight gain with an average of 25 g/day. There was a well-defined whitish peeling lesion with the shape of lips and it was compatible with the sucking pad. The lesion had started to desquamate like a peel (Figure 1). The mother also was trained for proper breastfeeding techniques to prevent sore nipples. Parents were reassured regarding the benign nature of the baby’s condition and the baby was discharged with a follow-up an arrangement at the Lactation Management Centre.

Case 2

A one-month-old first-born female child presented with recurrent peeling of lips since birth. This baby was born by a lower segment cesarean section due to past-dates and fetal distress following meconium-stained liquor. The baby had a good Apgar score at birth and the birth weight was 2.9 kg. The neonatal examination was normal, except for the sucking pad in the lips. Parents were reassured about the benign nature of the lesion and explained that was compatible with the sucking pad and it occurred in the intrauterine period and might recur even with vigorous sucking afterward. Sucking was established within half an hour and the baby was observed in the mother-baby unit for 24 hours and discharged home. After a week, the lesion on the lips peeled off. However, the mother observed that the lesion in the lips had recurred associated with pigmentation. It did not interfere with either feeding or activity. After the baby was seen by a general practitioner, the mother had been reassured that the baby had oral thrush and was advised to apply Candida oral gel. Since there was no improvement, the baby was brought to the pediatrician.

**Figure 2:**
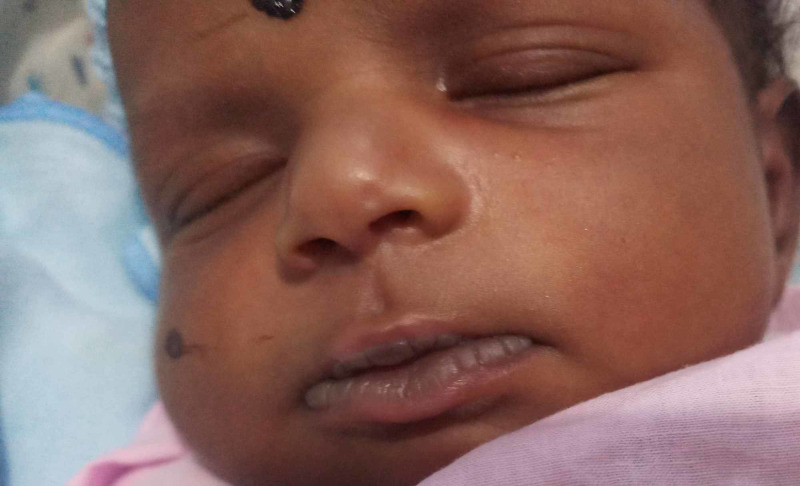
Well-developed sucking pad

Physical examination revealed that the baby was well-hydrated, active, and pink. The baby had good weight gain (30 g/day). Neonatal examination revealed that the baby had a whitish peel with a dark complexion (Figure 2). The rest of the examination was normal. Since it was compatible with the sucking pad, the mother was again reassured that it was a normal phenomenon, and arrangements were made to follow up at the Well-Baby Clinic for routine care. 

## Discussion

When the babies are delivered from aquatic intrauterine life to aerobic independent life, they acquire coordination of the nervous system, especially motor coordination, to have independent nasal breathing and breastfeeding. The sucking needs lots of modification of oral structures and intraoral pressure as a part of the extrauterine adaptation which subsequently disappears in later life [3-4]. The sucking mechanism operates a light movement from front to back on the relaxed tongue at the base of the mouth. Relative hypertrophy of the lip compressor muscle due to repeated tactile stimulation from the lips is the major mechanism for nipple gripping and sucking [4].

Sucking pads occur at the inner aspect of the vermillion (mucocutaneous junction) border of the lips due to an airtight seal and friction in terms of effective sucking which is facilitated by saliva and this region becomes hyperkeratotic and thickened as an adaptation mechanism [1, 4]. In other words, sucking pads occur immediately caudal to the closure of the line of the lips in neonates. They usually disappear after three to six months of age [4-5]. One report suggested that it persists as long as breastfeeding continues [1].

There were several differential diagnoses, including sucking blisters, which usually appear in areas accessible to the baby’s mouth, such as the thumb and arm. It might also be present at birth due to vigorous intrauterine sucking movements severe enough to form a sucking blister [5]. Oral thrush also sometimes mimics the sucking pad [4]. Another diagnosis confused with the sucking pad is leukoedema, which is a diffuse opalescent, non-peeling lesion of the buccal or labial mucosa which has been detected in newborns, teenagers, and also in adults [6-8]. Although there were no definite aetiologies suggested as the cause for leukoedema, possible reasons proposed include cheek biting and thumbsucking in babies [7]. Several explanations for leukoedema are suggested by various authors [7, 9].

The histology of the sucking pad is the same as leukoedema [5]. It has increased thickness of the epithelium (large edematous and poorly staining cells in the stratum and broad rete ridges of the affected area). The histologic appearance of a gradient effect, associated with the intracellular edema decreasing from the surface of the epithelium inward, is suggested by fluids (most likely saliva) which passively diffuses from the oral cavity into the mucosal cells. Also, keratin filaments are dispersed in the oedema fluids, while the rest of the cytoplasmic content, except nuclei, is compressed against cells [4].

Herein, we have summarized that the sucking pads are the combination of epithelial hyperplasia due to the effects of an adaptive mechanism of sucking and intracellular edema due to the effects of a pressure gradient that arises in the tissue as a result of the dynamic effects of sticking while sucking [1]. These pads are normal and fairly common anatomical variations. They contain labial vesicles or bullae filled with clear serous fluids that may rupture and healing occurs spontaneously. There is no therapy required for this benign condition [4-5].

## Conclusions

The sucking pad is a benign lesion in breastfed infants. Both preterm and term babies can have a sucking pad. It indicates effective sucking. It also acts as a neonatal screening tool. Since it is asymptomatic in all babies, it does not need intervention, except for explanation and reassurance.
